# Clinical presentations and biochemical profile in adult celiac disease patients in Hyderabad: Pakistan

**Published:** 2014

**Authors:** Naila Masood, Imran Ali Shaikh

**Affiliations:** 1 Dr. Naila Masood, MD, Associate Professor, Medical Unit-3,Liaquat University of Medical and Health Science (LUMHS), Jamshoro, Sindh, Pakistan.; 2 Dr. Imran Ali Shaikh, FCPS, Associate Professor, Medical Unit-3,Liaquat University of Medical and Health Science (LUMHS), Jamshoro, Sindh, Pakistan.

**Keywords:** Adult celiac disease, Hyderabad, Clinical Presentation

## Abstract

***Objective:*** To see the various clinical presentations and biochemical profile in adult celiac disease patients of Hyderabad Sindh.

***Methods:*** A total 60 suspected cases of adult celiac disease, both males and females were screened out from Liaquat University of Medical and Health Sciences hospital and private clinics at Sadar Hyderabad Sind by non probability purposive sampling during a period from July 2011 to December 2012.Age ranged between 18 to 55 Years. A detailed history and clinical examination was done. Patients already on gluten free diet, age <12years, tuberculosis or cancer of intestine/colon and patients of diabetes and thyroid disorder were excluded, while patients having positive ant tTG (value >15 iu/ml detected by ELISA) were included. The biochemical profile including serum albumin, calcium ,ferritin, SGPT, Alkaline phosphatase and Haemoglobin were estimated in central Diagnostic laboratory LUMHS by taking 10 cc centrifuged blood sample. The data was plotted on SPSS 16, mean and percentages were calculated.

***Results: ***All patients were divided in to three groups according to age. The most common group was 18-30 years; (mean, 23.5±5.6) comprised 56.6%. The commonest clinical presentation was diarrhoea in 50%, menstrual irregularity in 21%, walking problems 21%, undue fatigue in 15% and edema in 15%. P values calculated in quantitative variable of males and females. The p value was significant in between serum calcium (p 0.004), haemoglobin (p 0,004), serum ferritin (<0.005) and alkaline phosphatise (<0.005).

***Conclusion:*** This study showed that Adult celiac disease was present with entirely different clinical and biochemical profile in patients in this region.

## INTRODUCTION

Celiac disease (CD) is gluten – induced multisystem autoimmune disorder and small intestine is the primary target.^1^ It may occur at any age and its symptoms vary considerably.^2^ In children under two years of age gastrointestinal symptoms and failures to thrive are common. In older children and adults, symptoms are often nonspecific, such as abdominal pain, anemia, osteoporosis, fatigue, and even depression. Consequently, the diagnosis may easily be delayed or even missed.^3^ The etiology of CD is complex, where both genetic and environmental factors are involved. The human leukocyte antigen (HLA) genes encoding class II molecules i.e. DQ2 and DQ8 are risk factors. More than 90% of patients expressed DQ2 molecule while remaining carried DQ8 molecule.

The definitive diagnosis of CD was based on biopsy of small intestine that demonstrated increased number of intestinal lymphocytes, crypt hyperplasia and villous flattening which resolved on gluten – free diet.^4^ The disease has been well documented in Asians from India, Pakistan, and Iran.^5^ Using simple serological tests; it has gradually become clear that the prevalence of CD in different countries in the Middle East, North Africa, and India where wheat has been the major staple food for many centuries is almost the same as that in Western countries. Clinical studies showed that presentation with nonspecific symptoms or no symptoms is as common in the Middle East as it is in Europe. The CD is frequently unrecognized by physicians, in part because of its variable clinical presentation and symptoms.^6^ The recent reports have been shown that celiac disease is a common disorder in North Africa,^7^ the Middle East, India^8^ and Pakistan.^9^

A significant number of adult patients with celiac disease remain either silent or asymptomatic. In some reports celiac disease has been recognized as late as 70 years of age.^10^ Majority of the patients with atypical symptoms of celiac disease do not report to the hospitals in Pakistan. The “atypical form” of the disease is characterized by few, or no gastrointestinal. Symptoms. It predominantly presents with extra intestinal features such as neurological, Dermatological, hematological, endo crinological, reproductive, renal, psychiatric, skeletal, and liver involvement. Taddeucci et al conducted a study on 26 patients with CD and found that 31% were having abnormalities in central nervous system.^11^ The antigen against which EMAs are directed is tTG. The anti-tTG antibodies are highly sensitive and specific for the diagnosis of CD.^12^ Enzyme-linked immune sorbent assay (ELISA) tests for IgA anti-tTG antibodies are now widely available and are easier to perform, less observer-dependent, and less costly than the immunofluorescence assay used to detect IgA EMA antibodies.^13^

The clinical presentations are different in age groups such as shown by Santiago Vivas MD.^14^ They studied new cases of celiac disease diagnosed between 2000 and 2006 prospectively included (66 children and 54 adults). The clinical spectrum was categorized in two groups: (a) typical (malabsorption, chronic diarrhea, or failure to thrive) and (b) oligosymptomatic (abdominal pain, anemia, hyper transaminasemia). In the real sense worldwide, CD “out of the intestine” is 15 times more frequent than CD “in the intestine making the diagnosis extremely challenging.^15^ This study aimed to focus the clinical presentation and biochemical changes in adults suffering from celiac disease in Hyderabad. The awareness of celiac disease in this area is very little especially when celiac disease has been presented other than diarrhea.

## METHODS

Sixty patients suspected cases of adult celiac disease were screened out from Liaquat University of Medical and Health Sciences Hospital Hyderabad / Jamshoro and private clinics at Sadder, Hyderabad, Sindh by non probability purposive sampling, 22 were males (36.6.%) and 38 were females (63.3%).The age ranged between 18 to 55 years. The study was approved by ethical review committee of LUMHS.A written consent was taken from all the patients. A detailed history and clinical examination was done. Patients already on gluten free diet, age <12years, tuberculosis or cancer of intestine/colon and patients of diabetes and thyroid disorder were excluded, while patients having positive ant tTG (value >15 iu/ml detected by ELISA) were included. 

All groups had been sub categorized according to symptoms of initial presentation. The male to female differences noted according to biochemical profile. The main presentations were diarrhoea, menstrual irregularities, undue fatigue, walking difficulty and edema of feet. The biochemical profile including serum albumin, calcium ,ferritin, SGPT, Alkaline phosphatase and Haemoglobin were sent to central Diagnostic laboratory LUMHS by collecting 10 cc centrifuged sample. The data was plotted on SPSS 16, mean & percentage was calculated. The difference between male and female means in five biochemical parameters was calculated; serum albumin, calcium, haemoglobin serum ferritin and alkaline phosphatase. Two independent student t test were applied to observe mean difference in qualitative biochemical variables. P value was considered <0.005 significant.

## Results

 Out of 60 patients, 22(36.6%) were male and 38 (63.6%) were females. The age range was 18 to 55 years mean age was 38±10.5 years. All patients were categorized in to three groups according to age; group 1, 18-30 (56.6%) years mean age was23.5±5.6, 2^nd^ group, 31-42 Years (28.2%), mean age was 35.5± 3.5 and 3^rd^ group was in between 43-55 years (15%) mean was 47.6±4.2. The most common group was 18-30 years (mean 23.5±5.6) comprised 56.6% (Table-I). SPSS version 16 was used to analyze the data. Frequency and percentage was computed for categorical variables, mean and standard deviation (SD) for quantitative variables, independent student t test was used for biochemical parameters for p value. The clinical presentations were diarrhoea in 30 patients (50%). In the descending order menstrual irregularity in 13 patients, (21%), walking problems in 13 patients (21%), undue fatigue in 9 patients (15%) and edema in 9 patients (15%), abdominal bloating, fullness in 8 patients (13.3%), bony pain in 8 patients (13.3%), alopecia in 8 patients (13.3%) respectively (Table-II).

The biochemical parameters were measured statically which showed clear gender difference. Out of 7 biochemical parameters, anti tTG IgA were positive in both sexes equally. p value calculated which was significant in between serum albumin 3.4 ±1.28 and (3.2±1.3) p value was 0.006**,** serum calcium 8.0±1.8 and 7.9±1.4, p 0.004, haemoglobin 12.3±2.611.8±2.0 p 0.004, serum ferritin 23±10.217±9.45 p <0.005 and alkaline phosphatise 270±65.7 and 280±95.4 <0.005 (Table-III).

**Table-I:** Age groups (n=60).

*Age (years)* *Mean &SD*	*Male*	*Female*
* Group 1*	12(20%)	22(36.6%)
18-30 (23.5±5.6)		
*Group 2*	7(11.6%)	10(16.6%)
31-42 (35.3±3.5)		
*Group 3*	3(5%)	6(10%)
43-55 (47.6±4.2)		
*Total*	22	38

**Table-II:** Clinical presentation of patients (n=60).

*Clinical presentation*	*Groups*			*Percentage (%)*
	*1*	*2*	*3*	
Abdominal bloating	5	2	1	13.3
Diarrhoea	20	8	2	50
Bony pain	4	2	2	13.3
Undue fatigue	3	4	2	15
Menstrual problems	10	3	0	21.3
Alopecia	2	4	2	13.3
Walking difficulty	6	4	3	21.6
Edema over face & feet	4	2	3	15

**Table-III:** Biochemical parameters according to gender of patients ( n= 60).

*Biochemical parameters*	*`Male (22)*	*Female(38)*	*P value*
Anti tTG IgA	+ve	+ve	Ns
Serum albumin	2.6-4.5g/dl (3.4 ±1.28)	2.5-4.0g/dl (3.2±1.3)	0.006
Serum calcium	7.8-9.9mg/dl (8.0±1.8)	7.4-9.5mg/dl (7.9±1.4)	0.004
Haemoglobin	11.5-14.9g/dl 12.3±2.6	10.5-13.7g/dl 11.8±2.0	0.004
Serum ferritin	7-55mg/dl 23±10.2	3-45mg/dl 17±9.45	<0.005
Alkaline phosphates	50-400 i.u/L 270±65.7	40-450 i.u/L 280±95.4	<0.005
SGPT	10-70 i.u/L 27±6.5	15-65 i.u/ L 26±5.1	0.006

## DISCUSSION

This study was conducted in tertiary care hospital of Hyderabad Sindh. Total 60 patients were enrolled with clinical presentation of adult celiac disease in relation of biochemical parameters. In our study the female were dominated and the male to female ratio was 3:1(63 % vs. 22%). This ratio is matched with many national and international studies. Green et al^16^ enrolled 1612 patients from all United States and the women predominated (2.9:1). They also shown age of presentation in adult celiac disease could be as late as 5^th^ decade. The majority of respondents were diagnosed in their fourth to sixth decades.

In our study mean age was 37.8±12.5, out of 66 patients 65% were female and 34% were males and the main presentation was diarrhea in 50%. This ratio was lower than a study conducted in Hyderabad by Sadique^17^ et al where they found thirty patients (50/30) were female (60%) and mean age of participants was 33.25+ 9.7 years. Majority of (86%) patients presented with typical gastrointestinal symptoms. In their study they measures four clinical presentations while in our study we have 8 clinical presentations.

In our study, biochemical parameters are comparable to different studies conducted in different areas of world. We have found the serum albumin was 3.4g/dl ±1.28 and 3.2±1.3 and p value was 0.006**,** serum calcium 8.0 mg/dl±1.8 and 7.9mg/dl±1.4, p 0.004, haemoglobin 12.3 g/dl±2.611.8±2.0 p 0,004, serum ferritin 23mg/dl±10.217±9.45 p <0.005 and alkaline phosphatase 270iu/l±65.7 and 280iu/l±95.4 <0.005. Lionetti E et al^18^ demonstrated in their study Unexplained iron-deficiency anemia (3–15%). Green PH et al^19^ showed unexplained hypertransaminasemia (2–9%), Osteoporosis and osteomalacia of premature onset (2–4%) and recurrent abdominal pain or bloating.^20^

In our study the iron deficiency was present in both sexes. The patients commonly presented with undue fatigue in 15%. The serum iron levels were low and the mean value was 4.5mg/ dl. Our study matched with Sanders DS et al^21^ who has provided evidence to support atypical symptoms was 2.5 times more common than the classically described gastrointestinal presentation. In particular iron deficiency anemia accounted for 20.1% of all cases. They observed less than 5% patients with raised liver enzyme. In our study we have observed raised SGPT in 6 patients (10%). These raised levels in our patients were because they have used different drugs prescribed by general practitioners like analgesics, ATT, antidepressants. 

In our study 21% patients presented with different menstrual irregularities but we didn’t observe any reduction in fertility which is not match able to international studies such as carried out by Lewis^22^ et al who documented Infertility, reduced fertility, and an increased risk of an adverse outcome during pregnancy (miscarriage, low birth weight and intrauterine growth retardation) have all been attributed to undiagnosed celiac disease.

Andrew et al^23^ observed 1.9-16% ataxia of un known origin and alopecia in 2%. In our study walking difficulty was observed in 21% which is slightly higher than Andrew and the alopecia in 13% patients which is quite higher than above study. Our patients were on different medicines along with other nutritional deficiencies so alopecia was quite higher in our study.

## CONCLUSION

Celiac disease is presents in many ways. In adults the extra intestinal features are common such as undue fatigue, menstrual irregularities and walking difficulties. This study provides awareness of clinical and biochemical parameters of this important disease.

## Author Contribution:

***Naila Masood:*** Critical review and writing the final manuscript. 

***Imran Ali Shaikh:*** Collection of data, statical analysis and writing of manuscript.
